# Effects of Interactive Music Tempo with Heart Rate Feedback on Physio-Psychological Responses of Basketball Players

**DOI:** 10.3390/ijerph19084810

**Published:** 2022-04-15

**Authors:** Chung-Chiang Chen, Yi Chen, Li-Chuan Tang, Wei-Hua Chieng

**Affiliations:** 1 Office of Physical Education, National Yang-Ming Chiao-Tung University, Hsinchu City 300093, Taiwan; heroidf@yahoo.com.tw; 2 Department of Mechanical Engineering, College of Engineering, National Yang-Ming Chiao-Tung University, Hsinchu 300093, Taiwan; chani.me08g@nctu.edu.tw (Y.C.); newton4538.eo85g@nctu.edu.tw (L.-C.T.)

**Keywords:** rating of perceived exertion (RPE), heart rate feedback, interactive music tempo, beat per minute (BPM), basketball player, synchronous music, asynchronous music, sports-oriented music

## Abstract

This paper introduces an interactive music tempo control with closed-loop heart rate feedback to yield a sportsperson with better physio-psychological states. A total of 23 participants (13 men, 10 women; 16–32 years, mean = 20.04 years) who are professionals or school team members further guide a sportsperson to amend their physical tempo to harmonize their psychological and physical states. The self-tuning mechanism between the surroundings and the human can be amplified using interactive music tempo control. The experiments showed that listening to interactive music had a significant effect on the heart rate and rating of perceived exertion (RPE) of the basketball player compared to those listening to asynchronous music or no music during exercise (*p* < 0.01). Synchronized interactive music allows athletes to increase their heart rate and decrease RPE during exercise and does not require a multitude of preplanned playlists. All self-selected songs can be converted into sports-oriented music using algorithms. The algorithms of synchronous and asynchronous modes in this study can be adjusted and applied to other sports fields or recovery after exercise. In the future, other musical parameters should be adjusted in real-time based on physiological signals, such as tonality, beats, chords, and orchestration.

## 1. Introduction

Recently, the Olympic opening ceremonies, medal award ceremonies, and different competition events (i.e., gymnastics, figure skating) have often shown the prominent position of music in sports events, and music and sports have been increasingly associated [1]. Music has become an integral part of sporting events [2]. With the increase in recreational and individualized exercise programs, the incorporation of music into training has become more common. Several studies have focused on the potential benefits of music in sports. Many elite athletes listen to music during physical training sessions, pre-matches, and warm-ups because they believe that music can improve their mood, inspire them, and help them achieve their best performance level [3].

The influence of music on both psychology and exercise has been extensively studied. Three major factors (pitch, timbre, and rhythm of songs) affect the performance of various sports players [4,5]. Researchers usually use psychological scales, such as the rated perceived exertion (RPE) scale, to assess the fatigue of athletes and explore how their physiological and psychological responses and exercise performances are influenced by different musical elements [6]. The preference of music may mediate its motivational potential to a large extent, which shows that the choice of music has primary significance in determining the benefits that music may bring [4,7]. Furthermore, the influence of music is related to its internal elements, such as rhythm and musicality, and external factors arising from connections outside of culture and music [8,9]. In the application of music to sports, the tempo is considered the most important determinant of the response to music [10,11] and preferences for different tempos are influenced by the listener’s physiological arousal and the context in which the music is heard [12]. Notably, arousal intensity is highly positively correlated with heart rate response [13]. Experiments confirm that people’s preferred music tempo is positively correlated with their heart rate [14,15,16]. In this experiment, the researchers asked the participants to find their favorite tempo through self-regulation of a 440-Hz pure tone. As expected, the preferred tempo was close to the heart rate. To extend this to music stimulation, the relationship between the heart rate and music tempo preference was analyzed. The participants used a computer to control the tempo of the music. The results confirmed a significant positive correlation between preferred tempo and heart rate [14]. This suggests that fast tempo music may be preferred during physical activity, although some studies have suggested that slower tempos may increase physiological efficiency and thus prolong exercise performance [17,18].

Certain early experiments have revealed that music has a significant effect on the heart rate in humans [19], while the heart rate response is positively correlated with arousal level [13]. Listening to music before and during exercise can increase motivation and effort, thereby improving performance [20,21]. It can also improve endurance [20,22], sprinting [23,24], and resistance modes of exercise [21,25]. Basketball players have increased arousal levels in front of the audience and music groups and, thus, achieve better athletic performance [26,27,28]. Therefore, designated music responses to certain exercises can motivate athletes’ intentions and enhance their performance. For instance, music has been proven to effectively reduce fatigue and exertion through separation and distraction during exercise [29,30,31,32,33]. Performance improvement may be mediated by improved mood, exercise enjoyment, and increased feelings of power [4,23,34,35]. The increase in arousal and neural activity while listening to music has been shown to be accompanied by an improvement in exercise performance [34,36,37]. In addition to exercise, listening to music can help heart rate recovery after exercise [38]. In summary, the influence of music on exercise performance is a multifaceted topic involving various exercise modalities and may provide benefits for a wide range of athletic populations. However, some studies have shown that listening to music has little benefit to exercise performance, which is slightly different from the results of previous studies [39,40,41]. The attempts to identify the obvious factors and contexts in which music affects the physiology, psychology, and performance of sports and physical activities are still ongoing. In athletes, listening to music to reduce RPE is effective in low- and moderate-intensity exercise but evidently not in high-intensity exercise [41]. Music tempo may be a useful regulatory tool to prompt free-living individuals to reach an appropriate stride rate to achieve a walking pace of at least moderate intensity [42]. Some music is “activating” in the sense that it increases the speed, and some music is “relaxing” in the sense that it decreases the speed compared to the spontaneous walking speed in response to metronome stimuli. The participants were consistent in their observation of qualitative differences between relaxing and activating musical stimuli [43]. Instead of intensity-exercise and beat-walking interactions, this study focuses on the feedback music influences of exercise with both physiological and psychological factors, which are both HR and RPE.

The feedback or background music effects were extensively examined in this study as the physiological and psychological effects of asynchronous or synchronous music rather than the functional effects [6]. Synchronization of music tempo and exercise may improve the efficiency and overall performance of the exercise [44,45,46]. Such effects, physiological and psychological responses to synchronous music, have been demonstrated in bench stepping [47], cycle ergometry [48], callisthenic-type exercises [49], 400-m running [45], and multi-activity circuit tasks [50]. The sports brand Nike has also collaborated with the orchestra LCD Soundsystem under the American independent record company, Dairy Farmers of America Inc. (DFA). They collaborated to release the Original Run series music tailored for runners on iTunes in 2006, as a composition “45:33”, which is a song with the tempo based on the heart rate of the full cycle of jogging: from warm-up, stable peaks to slowly settling music [51]. Synchronous music tempo is discernably an important factor; therefore, this study focuses on how asynchronous or synchronous music tempo influences the physiological and psychological responses of athletes during exercise tasks.

The importance of body rhythm is well known in basketball performance [52], and listening to synchronized music while performing an exercise task appears to be helpful in heart rate response, physiological arousal, and RPE. To date, no study has focused on all musical elements being the same as control variables (all participants listened to the same song during the exercise task) and used heart rate feedback to control the tempo of the playing song as the independent variable to generate interactive music. The tempo algorithm is divided into synchronous and asynchronous based on the heart rate. This study compared the physical and psychological responses of basketball players with synchronous, asynchronous, or no music during sprints and technical tasks. The continuous real-time heart rate and RPE scale of the players were compared.

## 2. Materials and Methods

### 2.1. Study Design, Setting, Sample Size

#### 2.1.1. Participants

In total, there were 23 participants (13 men, 10 women; 16–32 years, mean = 20.04 years) in this study, including P. LEAGUE+ (PLG) (*n* = 1); Women’s Super-Basketball League (WSBL) (*n* = 5); National Yang Ming Chiao Tung University (NYCU) men’s basketball team, which includes sports gifted (*n* = 3) and general students (*n* = 4); High School Basketball League (HBL), which includes U17~U18 men’s team (*n* = 5) and U16 women’s team (*n* = 5). The following participant information was collected: gender, age, team, mass, height, BMI (body mass index, obtained by dividing the weight in kg of the participant by the square of the height expressed in meters), HR_rest_ (participants’ heart rate measured in a resting state), and HR_max_ (theoretical maximum heart rate) [53]. All participants were randomly divided into two groups. The demographics from this study are shown in Table 1. The method, experiment design, and safety of participants were strictly approved by the Research Ethics Committee for Human Subject Protection, National Yang Ming Chiao Tung University, before performing the experiments. The data collections were administrated by the author Chung-Chiang Chen, who is a professional basketball coach. In addition to this study, the data collections are also being used for basketball training courses and game tactical planning reference in the fourth quarter of 2021. All data collections were for research and teaching purposes only and absolutely no other use. The inclusion criteria for participants were the prospective players participating in the fourth quarter basketball tournament in Taiwan in 2021, invited by coach Chung-Chiang Chen. All participants regularly performed physical activity five times a week, and a good proportion of the participants were involved in heavy weight training. Participants provided written informed consent for the publication of this study. This experiment did not involve asking about health history, only whether participants were taking performance-enhancing, heart-rate-affecting illegal drugs that are banned from competition.

#### 2.1.2. Experimental Design

**Figure 1:**
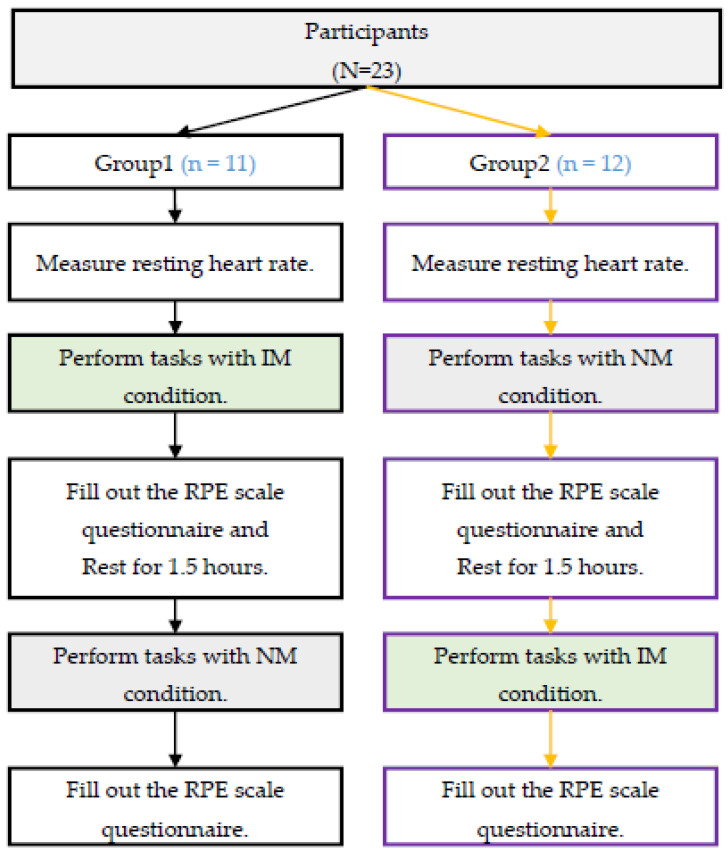
Counterbalanced Design.

In this study, the Quasi-Experimental Design method is employed, which is a type of experiment using all intact subjects to be given interactive music. The design is a quasi-experimental study with the type of “Counterbalanced Design” [54] (See Figure 1). The task has two conditions: no music (NM) and interactive music (IM). The physiological and psychological responses of participants performing the same task in the two conditions were compared using heart rate measurements and the RPE scale.

#### 2.1.3. Procedure

The detailed experimental procedure was conducted after explaining it to all the participants and receiving their consent. Smartwatches were worn by the participants, and their resting heart rates were measured before starting tasks. The task has two conditions, including no music (NM) and interactive music (IM). The experiments were randomly divided into two groups. For group 1, the task was first performed under IM conditions. After a 1.5-h rest, participants performed the task with the NM condition. For group 2, the task was to be performed under NM conditions first. After a 1.5-h rest, participants performed the task in the IM condition. Among them, the synchronous interactive music (SIM) was played when performing the shuttle runs to the fifth goal of the shooting task, and the asynchronous interactive music (AIM) was played from the fifth goal to the tenth goal in the shooting task. The heart rate was received via Bluetooth, the application records the raw data, and the time tags were observed and recorded by the researcher. The data collections were classified according to the time tags: sprint for 20 s, sprint for 40 s, sprint for 60 s, shoot 5 goals after the sprint, and shoot 10 goals after the sprint (See Table 2). As shown in the video in the Supplementary Materials Video S1, due to site constraints, this experiment only allowed two participants to perform the experiment at a time, and the participants spent approximately 5 min completing the two tasks. The time spent for 12 experiments was exactly 1 h. After the researchers completed the first round of experiments in each group, they confirmed that all participants’ questionnaires were filled out correctly. This process took 30 min before the second round of experiments began. This time spent constitutes the participant’s rest time.

#### 2.1.4. Exercise Tasks

**Figure 2:**
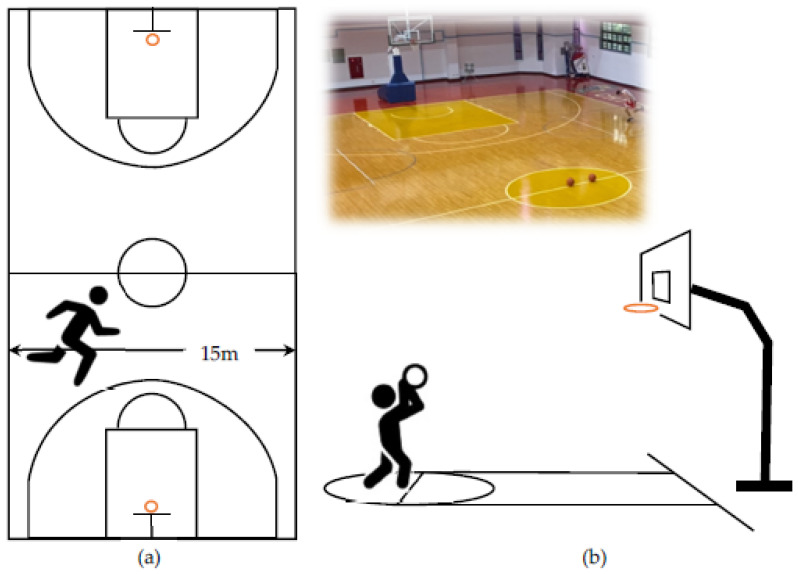
Tasks (**a**) Shuttle runs in 60 s (time-limited) and (**b**) Shoot 10 goals from the free-throw line (scores to achieve).

Each participant performed a shuttle run for a fixed time and then continued the two tasks with a fixed number of basketball shots. The participants performed shuttle runs on the narrow side of the basketball court (15 m) within 60 s, then shot the basketball from the free-throw line to score 10 goals (See Figure 2). During the shuttle run task, participants ran with their maximum power to achieve more laps within the time limit. During the free throw shooting task, players tried to complete 10 scored shoots in the shortest time possible.

#### 2.1.5. Sample Size

Based on the power analysis, this experiment had at least 20 participants in order to achieve the minimum of 80% power to reject the null hypothesis. The study used the Two-Way ANOVA: Repeated measures, within-between interactive design. The models are divided into 3 different sets, including 2 conditions × 1 factor (number of measurements = 2), 2 conditions × 2 factor (number of measurements = 4), and 2 conditions × 5 factor (number of measurements = 10). The maximum number of samples was required for the set of 2 conditions × 1 factor (number of measurements = 2). Given the design of the study, a power analysis conducted using G * Power 3.1.96 [55] requires simultaneously that the medium-to-large effect size f is lower than 0.35, α is lower than 0.05, and a power (1 − β) is higher than 0.80 when the number of groups is 2, the number of measurements is 2, and the sample size is 20.

#### 2.1.6. Devices and Data Collection Protocol

The Amazfit GTS 2 mini was used to measure the heart rate of participants during exercise through a smartwatch worn on the wrist. Heart rate measurements from wearables are derived from photoplethysmography (PPG), an optical method for measuring changes in blood volume under the skin. Although the accuracy of wearable optical heart rate measurers using PPG of the previous version has been questioned [56,57,58,59,60,61,62], the most updated literature from Brinnae Bent et al. concluded that different recent wearables are all reasonably accurate at resting and prolonged elevated heart rate [63]. Specifically, the Xiaomi device (with the same hardware as the Amazfit) used in this study performed well in accuracy during physical activity and was comparable to the experimental-grade device. During physical activity, the consumer-grade device Xiaomi had a mean absolute error (MAE) of 13.8 bpm, the research-grade device Biovotion had an MAE of 19.8 bpm, and the Empatica E4 had an MAE of 12.8 bpm [63].

In this study, the data collection from the NM, SIM, and AIM tests are the heart rates taken from the participants when they are either resting or during the prolonged elevated exercising stage. The data collected has been previewed to justify the correctness of the apparatus setting. The heart rate would be transmitted from every Amazfit GTS 2 mini via Bluetooth to the smartphone held by the data/booker or the participants. This study used Buds Air 2 Bluetooth headsets on smartphones to play music by the Nupiano app. Among them, the Bluetooth headset has the active noise reduction function enabled, and all participants were listening to the same music volume.

Whenever the participant’s heart rate changes, the wearable transmits it to the phone in real-time via Bluetooth. Amazfit software sends specific commands to a specific UUID to force it to measure the heart rate continuously, which receives the real-time heart rate by listening BluetoothGatt. The source code link is attached to the Supplementary Material File S1 and File S2, including the continuous measurement command and the UUID protocol.

#### 2.1.7. Data Collection

The time tags for every single trip of the participants in the shuttle run task and the time tags for each goal in the shooting task were recorded, and each raw data were labelled with device system time. The exercise performance of all participants was recorded, and physical and psychological responses at specific time points were stored.

##### Heart Rate Response

Studies have shown that arousal intensity is highly positively correlated with heart rate response [13]. In this study, both graphical observations and statistical analysis were used to assess heart rate responses. This study used time-stamped continuous heart rate raw data to create a heart rate response graphic and used the heart rate (HR) and average heart rate (aHR) data to estimate increases or decreases in arousal intensity. The heart rate data was classified according to the time tags and recorded as HR_20_, HR_40_, HR_60_, HR_5th_, and HR_10th_. Average heart rate data within the time tags interval were classified according to the time tags and recorded as aHR_20_, aHR_40_, aHR_60_, aHR_5th_, and aHR_10th_.

##### Rating of Perceived Exertion (RPE)

When the RPE scale was first proposed, it was a 15-point category ratio scale [64], ranging from 6 (very, very relaxing [rest]) to 20 (maximum exercise). It is used to measure the amount of self-perceived exercise during the task. The higher the degree of fatigue perceived in the task, the higher the RPE score. The RPE scale has proven to be closely related to physiological measurements (including heart rate). Since the scale of 6 to 20 points is not intuitive for the subjects, a new version of the scale of 0 to 10 points Borg CR10 Scale has been proposed, also known as Modified RPE [65,66]. This study uses the Borg CR10 scale as the questionnaire and analysis statistics. The questionnaire for this study was used to query the participants about the RPE in the first third, middle third, and final third of the shuttle run task, as well as the RPE of five goals in the shooting task after the shuttle run, and also the RPE from the fifth goal to the tenth goal, recorded as RPE_20_, PRE_40_, RPE_60_, RPE_5th_, and RPE_10th_.

##### Exercise Performance

The exercise task was divided into a shuttle run task and a shooting task. The performance of the shuttle run task was evaluated on the total number of trips (the more, the better), and the performance of the shooting task was evaluated by the goal time (a shorter time is better). The data collection was classified according to the time tags and recorded as Trips, Time_5th_, and Time_10th_.

### 2.2. Intervention

#### 2.2.1. Correlated Music Tempo with Heart Rate in BPM

**Figure 3:**
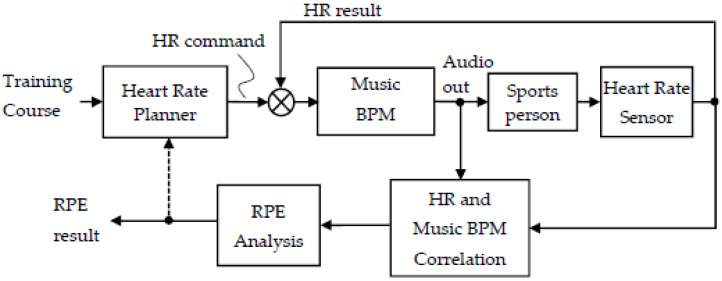
Correlated music tempo with heart rate (HR) in BPM.

This study designed a set of interactive music tempo control with a closed-loop heart rate feedback mechanism, as shown in Figure 3.

**Figure 4:**
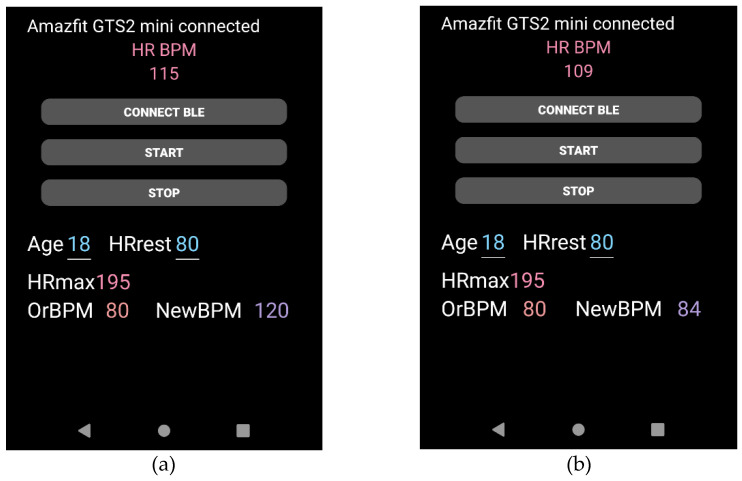
Two modes effects in the app, (**a**) Synchronous mode and (**b**) Asynchronous mode.

The Heart Rate Planner designed here in this experiment has two models: synchronous mode and asynchronous mode (See Figure 4). The detailed description is as follows:

For synchronous mode, the following equation is applied to update the music tempo:

(1) $$BPM_{music,new}=\left(1-e^{-\frac{HR_{measure}}{\left(HR_{max}-HR_{measure}\right)}}\right)\cdot \alpha \cdot BPM_{music,origin}$$

In the experiment of this study, *α* = 2.1, the chosen value, in Equation (1) makes the maximum music *BPM* equal to 80% *HR_max_* [53]. When the participant’s heart rate exceeds 80% of the maximum heart rate, it will become music *BPM* less than the heart rate. It is expected that this parameter design can relieve fatigue and increase sustained motivation during high-intensity exercise.

For asynchronous mode, the following equation is applied to update the music tempo:

(2) $$BPM_{music,new}=\frac{\beta \cdot HR_{rest}}{HR_{measure}}\cdot BPM_{music,origin}$$

The parameter in Equation (2), *β* = 1.5, is set up for the experiment. The original *BPM* of the music in this experiment is 76. Usually, the updated music *BPM* will be smaller than the measured heart rate *BPM*. However, when the measured heart rate is equal to the resting heart rate, which is measured when the participant is calm before the test, the updated music *BPM* will reach the maximum value, 1.5 times larger than the original music *BPM*. This asynchronous model can maintain a certain range through the music tempo no matter whether the measured heart rate is too low or too high.

#### 2.2.2. Nutext and Nupiano Player

**Figure 5:**
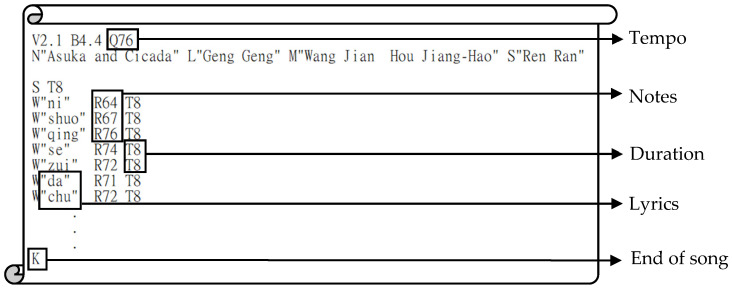
The Nutext format example.

Nupiano is a pure piano instrumental music player in the Nutext format. The Nutext format was designed based on numbered musical notation and follows the rules of numerical control codes. Nutext is similar to G-code. Its actions and events progress with time sequence, which is suitable for players that need to change the music tempo instantly [67]. An example of the Nutext format is shown in Figure 5, where the digits after Q are the tempo of this song in BPM. For the user, the tempo of the music can be changed only by changing the Q value in the program. Nupiano can easily change the playing tempo, and compared to audio-based players, it will not cause sound quality distortion due to the changing tempo. The source code link is attached in the Supplementary Material File S3, which contains synchronous mode and asynchronous mode algorithms.

#### 2.2.3. Music Selection

A study by Marc Leman et al. found that listeners and players share, to a certain degree, a sensitivity for musical expression and its associated corporeal intentionality [68]. Participants listening to the same song may perceive the same expressions and intentions. In this study, the music preferences of the participants were not considered, but the popular Chinese songs in the key of B flat major, which have become popular in recent years, were directly selected as control variables. “Asuka and Cicada” is the most popular Chinese song on the TikTok platform in 2020, and it also ranked No.1 in Taiwan’s PARTYWORLD list of request songs in 2020. The song is 76bpm of rhythm with B flat major. Pauer’s key characteristics for the B flat major are that it is “a favorite key of our classical composers, has an open, frank, clear, and bright character, which also admits the expression of quiet contemplation” [69].

### 2.3. Comparison

The participants did not wear headphones to perform exercise tasks, and no music was played on the experimental site.

### 2.4. Statistical Analysis

**Figure 6:**
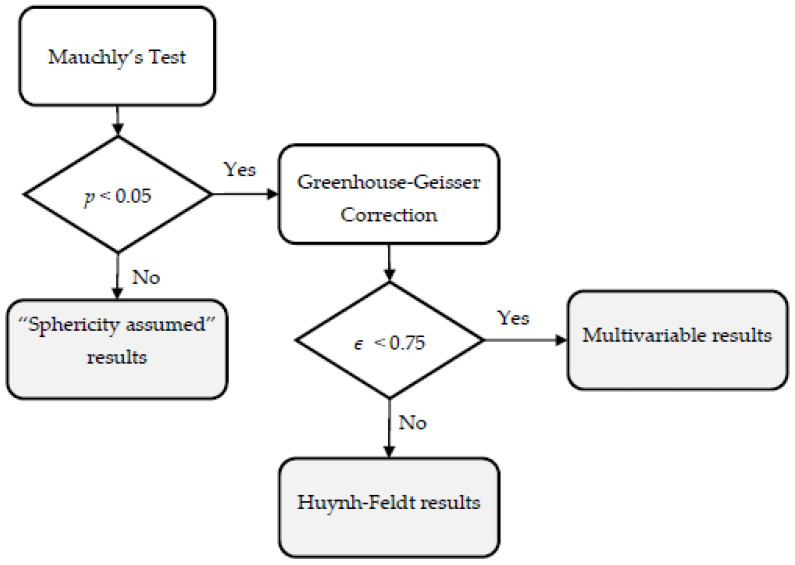
RM-ANOVA spherical flowchart.

Analyses were conducted using IBM SPSS Statistics Version 21.0 (IBM Corp., Armonk, NY, USA). The Shapiro–Wilk test was used to evaluate the normality of the data distribution. Successively, an analysis of variance with two-way repeated measures (RM-ANOVA) was conducted to determine whether significant differences existed between the two different conditions. This was considered the factor of the analysis (named Condition). A comparison of the heart rate (HR), average heart rate (aHR), RPE, Trips, and Goal-Time of basketball players listening to interactive music (including synchronous mode and asynchronous mode) and not listening to music at different time points when performing exercise tasks was conducted. The data collections were classified according to the time tags: sprint for 20 s, sprint for 40 s, sprint for 60 s, shoot 5 goals after the sprint, and shoot 10 goals after the sprint. Three sets of statistical models were used in this study, as shown in Table 3, including a series of 2 (Condition: NM, IM) × 5 (Time Tags) × 2 (Gender) mixed-model repeated-measures analysis of variance (RM-ANOVA) conducted on HR, aHR, and RPE for each exercise, a series of 2 (Condition: NM, IM) × 1 (Trips) × 2 (Gender) mixed-model repeated-measures analysis of variance (RM-ANOVA) conducted on Trips for the shuttle run task, and a series of 2 (Condition: NM, IM) × 2 (Time Tags) × 2 (Gender) mixed-model repeated-measures analysis of variance (RM-ANOVA) conducted on Goal-Time for the shooting task. All the variables were transferred into the Within-Subjects Variables: (Condition, HR), (Condition, aHR), (Condition, Trips), (Condition, Goal-Time). Huynh-Feldt correction applied to all RM-ANOVAs if they violated the spherical assumption. The RM-ANOVA report follows the spherical flow chart of previous rules of thumb for statistical research in psychology [70,71], as shown in Figure 6.

## 3. Results

Descriptive data for HR, aHR, RPE, Trips, and Goal-Time for each condition and at each measurement point are presented in Table 4.

**Figure 7:**
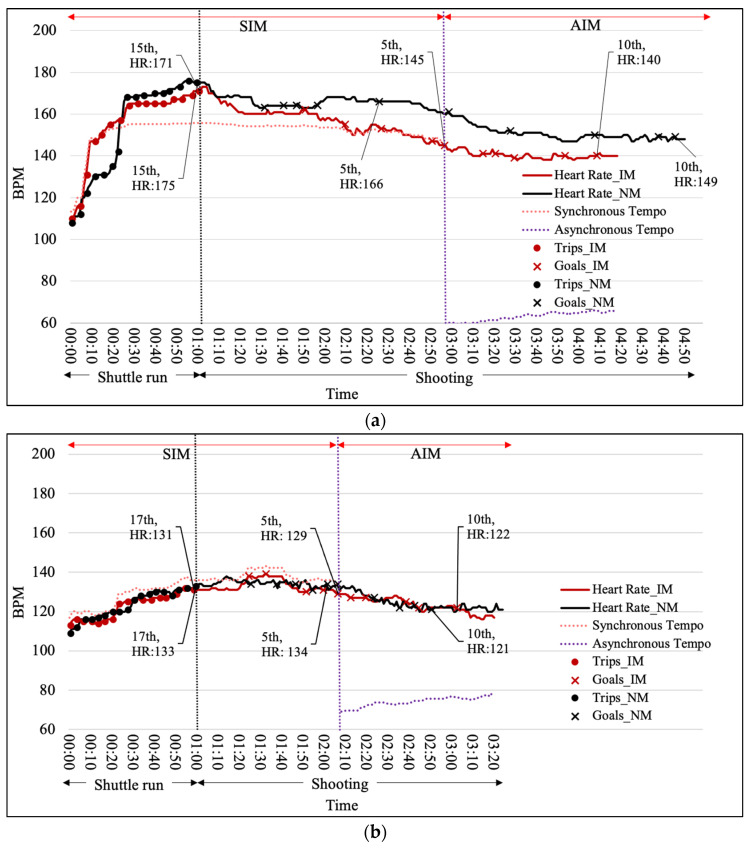
Heart Rate Response during the tasks with IM and NM conditions (**a**) could be clearly observed (**b**) could not be clearly observed.

While interpreting the experimental results with the observation of red and black lines in the heart rate response graphs, it was found for some participants that the heart rate response with the IM condition (red line) and the NM condition (black line) could be clearly observed to be different (see Figure 7). A total of 10 participants’ (43.48%) heart rate response graphs were clearly observed to differ between the IM condition (red line) and the NM condition (black line).

### 3.1. Interaction Effects: Condition × HR, Condition × HR × Gender

**Figure 8:**
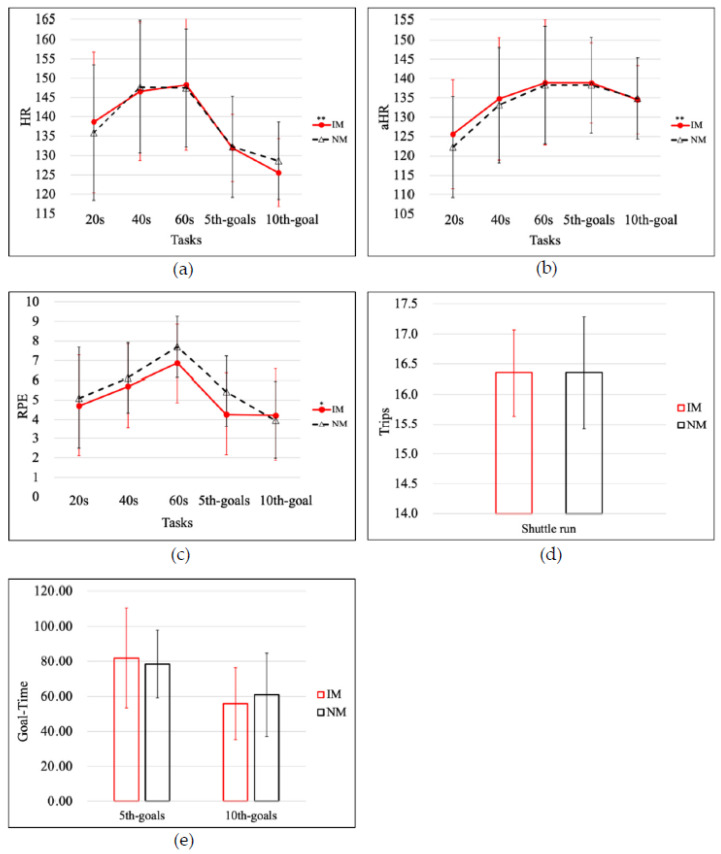
(**a**) The heart rate response, (**b**) the average heart rates (aHR) response and (**c**) the RPE response during the tasks with IM (red) and NM (black). (**d**) The shuttle run task performance and (**e**) the shooting task performance. * *p* < 0.05; ** *p* < 0.01.

There was a very significant difference in HR as a function of the interaction of Condition × HR with *p* = 0.004 and *η*^2^ = 0.56. There was no significant difference in HR as a function of the interaction of Condition × HR × Gender with *p* = 0.329 and *η*^2^ = 0.05. The results obtained for each individual condition are depicted in Figure 8a. Regardless of gender, basketball players listening to interactive music while performing exercise tasks always had significant effects on HR.

### 3.2. Interaction Effects: Condition × aHR, Condition × aHR × Gender

There was a very significant difference in aHR as a function of the interaction of Condition × aHR with *p* = 0.003 and *η*^2^ = 0.58. There was an insignificant difference in ΔHR as a function of the interaction of Condition × ΔHR × Gender with *p* = 0.907 and *η*^2^ = 0.01. The results obtained for each individual condition are depicted in Figure 8b. Regardless of gender, basketball players listening to interactive music while performing exercise tasks always had significant effects on aHR.

### 3.3. Interaction Effects: Condition × RPE, Condition × RPE × Gender

There was a significant difference in RPE as a function of the interaction of Condition × RPE with *p* = 0.014 and *η*^2^ = 0.48. There was no significant difference in RPE as a function of the interaction of Condition × RPE × Gender with *p* = 0.741 and *η*^2^ = 0.02. The results obtained for each individual condition are depicted in Figure 8c. Regardless of gender, basketball players listening to interactive music while performing exercise tasks always had significant effects on RPE.

Among the 23 participants, 14 participants (60.87%) had a RPE_5th_ score with the SIM condition (RPE_20_: 4.70 ± 2.58, RPE_40_: 5.70 ± 2.16, RPE_60_: 6.87 ± 2.01, RPE_5th_: 4.26 ± 2.09) lower than the NM condition (RPE_20_: 5.09 ± 2.59, RPE_40_: 6.13 ± 1.79, RPE_60_: 7.70 ± 1.58, RPE_5th_: 5.43 ± 1.81), and 19 participants (82.61%) had a RPE_10th_ score with the AIM condition (RPE_10th_: 4.22 ± 2.35) higher than the NM condition (RPE_10th_: 3.96 ± 1.99). Listening to synchronous music tempo while performing exercise tasks was helpful in reducing RPE while listening to asynchronous music tempo hardly compared to not listening to any music.

### 3.4. Interaction Effects: Condition × Trips, Condition × Trips × Gender

There was not a significant difference in performance as a function of the interaction of Condition × Trips with *p* = 0.829 and *η*^2^ = 0.00. Neither was there a significant difference in Trips as a function of the interaction of Condition × Trips × Gender with *p* = 0.109 and *η*^2^ = 0.12. The results obtained for each individual condition are depicted in Figure 8d. Regardless of gender, basketball players listening to interactive music while performing exercise tasks had no significant effect on sprint exercise performance.

### 3.5. Interaction Effects: Condition × Goal-Time, Condition × Goal-Time × Gender

There was no significant difference in performance as a function of the interaction of Condition × Goal-Time with *p* = 0.332; *η*^2^ = 0.05. There was also no significant difference in Goal-Time as a function of the interaction of Condition × Goal-Time × Gender with *p* = 0.479 and *η*^2^ = 0.02. The results obtained for each individual condition are depicted in Figure 8e. Regardless of gender, basketball players who listened to interactive music while performing exercise tasks experienced no significant effect on shooting exercise performance.

The experimental results also indicated that listening to synchronous interactive music to perform exercise tasks has a very significant impact on increasing the heart rate response (*p* < 0.01), but with the increase in exercise intensity and RPE, the effect seems to be less obvious, as shown in Figure 8. Regardless of the sprinting or shooting tasks, the RPE scale of athletes listening to synchronous interactive music was lower than that of no music and asynchronous interactive music. The conclusion was statistically significant (*p* < 0.05). Listening to synchronous interactive music or asynchronous interactive music did not appear to have a significant effect on athletic performance when athletes performed exercise tasks.

## 4. Discussion

Conclusions can be drawn from the analysis in the previous sections. The basketball player would have an increased heart rate response resulting in an increase in physiological arousal intensity and a decrease in RPE when listening to synchronized interactive music during sprinting and shooting. However, it was not much gain in physiological efficiency compared to the exercises performance [17,18] when listening to slow tempo asynchronous interactive music. It seems that listening to slow-tempo asynchronous interactive music to perform exercise tasks did not seem to help heart rate response and RPE. Listening to synchronous interactive music versus slow tempo asynchronous interactive music to perform exercise tasks made no difference in sprint or shooting performance. Basketball is a physical rhythm-focused sport, and during the exercise, the athlete’s heart rate reaches 80% of the theoretical maximum heart rate, and the body rhythm is an important factor in shooting [52]. Based on these theories, if the basketball player listens to music in sync with their own body rhythm while exercising, their heart rate response will tune to achieve the physiological arousal levels and, thus, RPE is reduced. Therefore, the results of this study may explain why the PRE of basketball players listening to slow tempo asynchronous interactive music for exercise tasks is higher than that of no music and synchronous interactive music. As listening to slow tempo asynchronous interactive music does not help with physiological arousal intensity, it takes more effort for a basketball player to achieve the level of arousal in the shooting state.

In contrast to the current study, athletes listening to fast-tempo music (>120 BPM) during sprints had a significant effect on heart rate, but not sprint performance, as in previous studies [23,24]. Notably, the results of this study appear to have a more significant effect on heart rate increases in the first 20 s of sprint initiation, with listening to synchronized interactive music significantly reducing RPE regardless of the exercise task. Previous research indicated that listening to the music of self-choice while exercising resulted in lower RPE in women, but it did not seem to help men [24]. This is in contrast to the current study, where there was no significant difference in RPE between the genders of the participants. All participants listening to synchronized music while performing exercise tasks had lower RPE; the result is also statistically significant (*p* < 0.05). Previous studies had shown that listening to fast-tempo music while basketball players warmed up could significantly increase heart rate and improve the athlete’s level of arousal, thereby improving athletic performance [28]. In contrast to the current study, listening to synchronized music during exercise tasks had a significant effect on increased heart rate responses, but appeared to have no effect on exercise performance.

Physical activation is very important for feeling tired because the signals transmitted from the body to the brain inform the brain of the ongoing efforts, thereby regulating physical activity. These signals capture conscious attention and change behavioral responses relating to exercise adherence [33]. However, music can be considered a useful tool for regulating the intensity of physiological arousal and subjective experience to increase the level of physical activity and sports participation [4,23,30,31,32,33,34,35,36,37]. Music is strategically chosen to elicit physical and psychological responses for better performance, experience, and persistence during exercise, as well as to regulate emotions and distract attention from discomfort [4,7]. The biggest difference between this research and the previous study is that this study can adjust the music parameters according to the heart rate in real-time to induce physio-psychological responses. Through the mechanism of this study, there is no need to have a large number of playlists or conduct playlist planning in advance. In addition, sportspeople can also choose music according to their own preferences, which can all be turned into sports-oriented music through algorithms.

## 5. Conclusions

This study successfully introduced the interactive music tempo control with a closed-loop heart rate feedback mechanism to realize synchronous and asynchronous music experiments with the same music element as the control variable (same song). During the shuttle run and shooting tasks in the experimental results, listening to interactive music had a significant effect on the heart rate (Condition × HR), average heart rate (Condition × aHR), and RPE (Condition × RPE) of the basketball player; the result is also statistically significant (*p* < 0.05). Among them, the heart rate in the first 20 s of listening to the synchronous music sprint was significantly higher than that without music (HR_20_ with SIM: 138.65 ± 18.25 BPM; HR_20_ with NM: 135.91 ± 17.51 BPM), and the overall RPE was significantly lower than that of asynchronous music and no music. This means that basketball players listening to synchronous music have increased arousal and decreased RPE than asynchronous music and no music during exercise tasks. The contribution of this study lies in the ability to add personalized music that is close to the rhythm of the body during basketball training so that basketball players can better feel the rhythm of the body. The music helped basketball players to adjust the rhythm of their movements. There were 60.87% of players’ RPE values significantly lowered in exercise tasks with SIM condition; the results show that with SIM (RPE_20_: 4.70 ± 2.58, RPE_40_: 5.70 ± 2.16, RPE_60_: 6.87 ± 2.01, RPE_5th_: 4.26 ± 2.09) and NM (RPE_20_: 5.09 ± 2.59, RPE_40_: 6.13 ± 1.79, RPE_60_: 7.70 ± 1.58, RPE_5th_: 5.43 ± 1.81). In particular, the affection for the asynchronous mode of the interactive music tempo did not lower the RPE values obviously. In contrast, the RPE values of 82.61% of participants were raised with AIM condition (the results show that with AIM (RPE_10th_: 4.22 ± 2.35) and NM (RPE_10th_: 3.96 ± 1.99)). According to these results, it is possible to positively improve the basketball players’ arousal and lower fatigue when they receive a custom-made music tempo interaction scheme in their training courses. The mechanism of this study does not require a large number of playlists and pre-planned playlists, it just needs sportspeople to choose their favorite songs, and all songs can be turned into sports-oriented music through algorithms. In addition, the parameters and variables of our synchronous mode and asynchronous mode in this article can be adjusted and applied to more sports fields, or post-exercise recovery, even in addition to synchronized music based on heart rate. In the future, it can also be discussed to adjust other musical parameters in real-time based on physiological signals, such as tonality, beats, chords, orchestration, etc. The scope of application to recreational sports is not limited to professional sports training courses.

## Figures and Tables

**Table 1:** Demographics of participants.

	Group 1 (*n* = 11)			Group 2 (*n* = 12)			Overall
Gender	M (*n* = 6)	F (*n* = 5)	M/F 6/5	M (*n* = 7)	F (*n* = 5)	M/F 7/5	M/F 13/10
Age (years)	20.33 ± 5.79	19.80 ± 3.83	20.09 ± 4.76	19.71 ± 2.21	20.40 ± 6.19	20.00 ± 4.09	20.04 ± 4.32
Height (cm)	183.00 ± 7.21	170.60 ± 6.23	177.36 ± 9.14	184.14 ± 6.91	162.60 ± 5.81	175.17 ± 12.71	176.22 ± 10.95
Mass (kg)	76.00 ± 6.48	61.78 ± 5.02	69.54 ± 9.29	77.57 ± 9.57	55.48 ± 5.37	68.37 ± 13.78	68.93 ± 11.60
BMI (kg/m^2^)	22.70 ± 1.49	21.24 ± 0.94	22.04 ± 1.43	22.81 ± 1.73	20.94 ± 0.91	22.03 ± 1.69	22.03 ± 1.54
HR_rest_ (BPM)	78.67 ± 5.28	73.60 ± 9.61	76.36 ± 7.61	78.86 ± 6.28	78.60 ± 4.56	78.75 ± 5.40	74.09 ± 6.51
HR_max_ (BPM)	193.33 ± 4.18	193.60 ± 2.51	193.45 ± 3.36	194.00 ± 1.53	193.00 ± 4.24	193.58 ± 2.84	193.52 ± 3.03

**Table 2:** Procedure.

Phase	Condition	Data Collection with Time Tag	Rules
Pre-task	SIM NM	HR_rest_	10 min
Shuttle run	SIM NM	HR_20_/RPE_20_ HR_40_/RPE_40_ HR_60_/RPE_60_ Real-time HR	60 s
Shooting I	SIM NM	HR_5th_/RPE_5th_ Real-time HR	5 goals
Shooting II	AIM NM	HR_10th_/RPE_10th_ Real-time HR	5 goals

**Table 3:** Affected factors and statistical models.

Effected Factors	Models
HR, aHR, RPE	2 (Condition: NM, IM) × 5 (Time Tags) × 2 (Gender) mixed-model
Trips	2 (Condition: NM, IM) × 1 (Trips) × 2 (Gender) mixed-model
Goal-Time	2 (Condition: NM, IM) × 2 (Time Tags) × 2 (Gender) mixed-model

**Table 4:** Descriptive statistics for HR, aHR, RPE, Trips, and Goal-Time during tasks with IM and NM conditions.

Condition	No Music (NM)		Interactive Music (IM)	
	Mean	Standard Deviation	Mean	Standard Deviation
HR_20_	135.91	17.51	138.65	18.25
HR_40_	147.65	16.99	146.52	17.75
HR_60_	147.43	15.18	148.26	16.86
HR_5th_	132.22	13.05	131.96	8.66
HR_10th_	128.65	10.06	125.57	8.76
aHR_20_	122.29	13.08	125.59	14.06
aHR_40_	133.13	14.96	134.74	15.81
aHR_60_	138.31	15.18	138.88	15.98
aHR_5th_	138.30	12.34	138.87	10.43
aHR_10th_	134.81	10.53	134.47	8.84
RPE_20_	5.09	2.59	4.70	2.58
RPE_40_	6.13	1.79	5.70	2.16
RPE_60_	7.70	1.58	6.87	2.01
PRE_5th_	5.43	1.81	4.26	2.09
RPE_10th_	3.96	1.99	4.22	2.35
Trips	16.35	0.94	16.35	0.71
Time_5th_	78.44	19.41	82.01	28.65
Time_10th_	60.97	23.85	55.86	20.54

## Data Availability

The raw data supporting the conclusions of this article will be made available by the authors, without undue reservation.

## Supplementary Materials

The following supporting information can be downloaded at: https://github.com/chani1206/interactive-HR-music, File S1: HeartRateService.java; File S2: CustomBluetoothProfile.java; File S3: Fragment3.java; Video S1: ExperimentProcedure.mp4 (accessed on 8 April 2022).
